# Systemic Homologous Neutralizing Antibodies Are Inadequate for the Evaluation of Vaccine Protective Efficacy against Coinfection by High Virulent PEDV and PRRSV

**DOI:** 10.1128/spectrum.02574-21

**Published:** 2022-03-22

**Authors:** Ming Qiu, Shubin Li, Mengxue Ye, Jixiang Li, Zhe Sun, Xinshuai Li, Yulin Xu, Yanzhao Xiao, Chen Li, Binghui Feng, Hong Lin, Wanglong Zheng, Xiuling Yu, Kegong Tian, Jianzhong Zhu, Nanhua Chen

**Affiliations:** a College of Veterinary Medicine, Yangzhou University, Yangzhou, Jiangsu, China; b Joint International Research Laboratory of Agriculture and Agri-Product Safety, Yangzhou, Jiangsu, China; c Comparative Medicine Research Institute, Yangzhou University, Yangzhou, Jiangsu, China; d Jiangsu Key Laboratory of Zoonosis/Jiangsu Co-Innovation Center for Prevention and Control of Important Animal Infectious Diseases and Zoonoses, Yangzhou, Jiangsu, China; e National Research Center for Veterinary Medicine, Luoyang, Henan, China; Oklahoma State University, College of Veterinary Medicine

**Keywords:** porcine epidemic diarrhea virus (PEDV), porcine reproductive and respiratory syndrome virus (PRRSV), chimeric vaccine, systemic homologous neutralizing antibodies (shnAbs), protective efficacy, neonatal piglet

## Abstract

G2 porcine epidemic diarrhea virus (G2 PEDV) and highly pathogenic porcine reproductive and respiratory syndrome virus 2 (HP-PRRSV2) are two of the most prevalent swine pathogens in China’s swine herds, and their coinfection occurs commonly. Several PED and PRRS vaccines have been utilized in China for decades, and systemic homologous neutralizing antibodies (shnAbs) in serum are frequently used to evaluate the protective efficacy of PED and PRRS vaccines. To develop a vaccine candidate against G2 PEDV and HP-PRRSV2 coinfection, in this study, we generated a chimeric virus (rJSTZ1712-12-S) expressing S protein of G2 PEDV using an avirulent HP-PRRSV2 rJSTZ1712-12 infectious clone as the viral vector. The rJSTZ1712-12-S strain has similar replication efficacies as the parental rJSTZ1712-12 virus. In addition, animal inoculation indicated that rJSTZ1712-12-S is not pathogenic to piglets and can induce shnAbs against both G2 PEDV and HP-PRRSV2 isolates after prime-boost immunization. However, passive transfer study in neonatal piglets deprived of sow colostrum showed that rJSTZ1712-12-S-induced shnAbs may only decrease PEDV and PRRSV viremia but cannot confer sufficient protection against dual challenge of high virulent G2 PEDV XJ1904-34 strain and HP-PRRSV2 XJ17-5 isolate. Overall, this study provides the first evidence that shnAbs confer insufficient protection against PEDV and PRRSV coinfection and are inadequate for the evaluation of protective efficacy of PED and PRRS bivalent vaccine (especially for the PED vaccine).

**IMPORTANCE** Porcine epidemic diarrhea virus (PEDV) and porcine reproductive and respiratory syndrome virus (PRRSV) coinfection occurs commonly and can synergistically reduce feed intake and pig growth. Vaccination is an effective strategy utilized for PED and PRRS control, and systemic homologous neutralizing antibodies (shnAbs) in serum are commonly used for protective efficacy evaluation of PED and PRRS vaccines. Currently, no commercial vaccine is available against PEDV and PRRSV coinfection. This study generated a chimeric vaccine candidate against the coinfection of prevalent PEDV and PRRSV in China. The chimeric strain can induce satisfied shnAbs against both PEDV and PRRSV after prime-boost inoculation in pigs. But the shnAbs cannot confer sufficient protection against PEDV and PRRSV coinfection in neonatal piglets. To the best of our knowledge, these findings provide the first evidence that shnAbs confer insufficient protection against PEDV and PRRSV coinfection and are inadequate for evaluating PED and PRRS bivalent vaccine protective efficacy.

## INTRODUCTION

Porcine epidemic diarrhea (PED) is a highly contagious enteric disease principally causing diarrhea, vomiting, dehydration, and high mortality in suckling piglets, while porcine reproductive and respiratory syndrome (PRRS) is another highly contagious disease characterized by reproductive failure in sows and respiratory disease in all ages of pigs (1–3). In China, PED was first confirmed in 1984 and PRRS was first reported in 1995 (4, 5). Within the large number of viral diseases prevalent in China, PED and PRRS are proposedly two of the most economically significant viral diseases in China’s swine industry in decades.

PED virus (PEDV) is an enveloped, positive-sense, single-stranded RNA virus belonging to the family of *Coronaviridae* in the order of *Nidovirales* (6). According to antigenic and immunogenetic differences, PEDV strains can be classified into two groups: G1 and G2. In China, PEDV isolates before 2010 were clustered into G1, whereas PEDV variants after 2010 were mainly grouped in G2 (7). PEDV genome is ∼28 kb containing 5′ and 3′ untranslated regions (UTRs) and at least seven open reading frames (ORFs), which encode two replicase polyproteins (pp1a and pp1b), four structural proteins (spike [S], envelope [E], membrane [M], and nucleocapsid [N]), and a hypothetical accessory protein (2). The S glycoprotein is on the surface of a viral particle, which interacts with host cellular receptor to mediate viral entry. The S protein contains at least four neutralizing epitopes, which are commonly used as a target antigen for vaccine development (8).

PRRSV virus (PRRSV) is also a positive-sense, single-stranded RNA virus, which is grouped in the family of *Arteriviridae* within the order of *Nidovirales* (1). PRRSV can be divided into two species: PRRSV1 and PRRSV2. In China, PRRSV1 isolates were sporadically detected and caused endemics in recent years (9–11), whereas PRRSV2 isolates were predominant and frequently caused outbreaks (5, 12–14). Highly pathogenic PRRSV2 (HP-PRRSV2), NADC30-like PRRSV2 and NADC34-like PRRSV2 variants are currently the most prevalent isolates in China’s swine herds (15, 16). Nowadays, PEDV and PRRSV are two of the most prevalent swine viruses in China. Moreover, the coinfection of PEDV and PRRSV occurs commonly, which may reach 33.3% (17).

Vaccination is a common strategy used for combating PED and PRRS in China. But both inactivated and modified live vaccines (MLV) derived from G1 CV777 strain become insufficiently protective due to the emergence of G2 PEDV variants in China in October 2010 (18, 19). Several commercial PRRS MLVs are also manufactured and widely utilized in China (20). However, no vaccine is available for combatting the dual infection of prevalent PEDV and PRRSV isolates. The intensive immunization schedules containing distinct MLVs against different pathogens not only increase the cost of vaccination, but also become a big pressure to host immune system.

Neutralizing antibodies (nAbs) are vital components of the immune armory against viral infection (21, 22). S protein of PEDV is the main inducer of nAbs, whereas minor glycoproteins (GP2a, GP3, and GP4) and major structural proteins (GP5 and M) of PRRSV contain multiple neutralizing epitopes (21, 23–26). Systemic homologous nAbs (shnAbs) in serum are commonly used to evaluate the protective efficacy of PED and PRRS vaccine candidates. For instance, chimeric transmissible gastroenteritis virus (TGEV) and vesicular stomatitis virus (VSV) expressing S protein of G2 PEDV could induce shnAbs and provide protection against virulent G2 PEDV challenge (8, 27). In addition, chimeric PRRSV strains containing mixed ORF5-6 genes, shuffled ORF3-6 genes, or consensus ORF2-6 sequence could even induce heterologous snAbs and conferred cross-protection against heterologous PRRSV strains (3, 28, 29). However, whether PEDV and PRRSV shnAbs could confer dual protection against the widely prevalent highly pathogenic G2 PEDV and HP-PRRSV2 isolates in China has not been described yet.

Previous studies confirmed that PRRSV infectious clones could be used as effective live vectors for foreign gene expression (30–33). To develop a vaccine candidate against G2 PEDV and HP-PRRSV2 coinfection, we constructed a chimeric HP-PRRSV2 strain expressing S protein of G2 PEDV in this study. The *in vitro* and *in vivo* replication efficacies of this chimeric virus were determined. In addition, the safety and immunogenicity were evaluated by pig inoculation. Furthermore, the protective efficacy was assessed by passive transfer and challenge studies in neonatal piglets.

## RESULTS

### The chimeric rJSTZ1712-12-S virus can express S protein of G2 PEDV.

**FIG 1:**
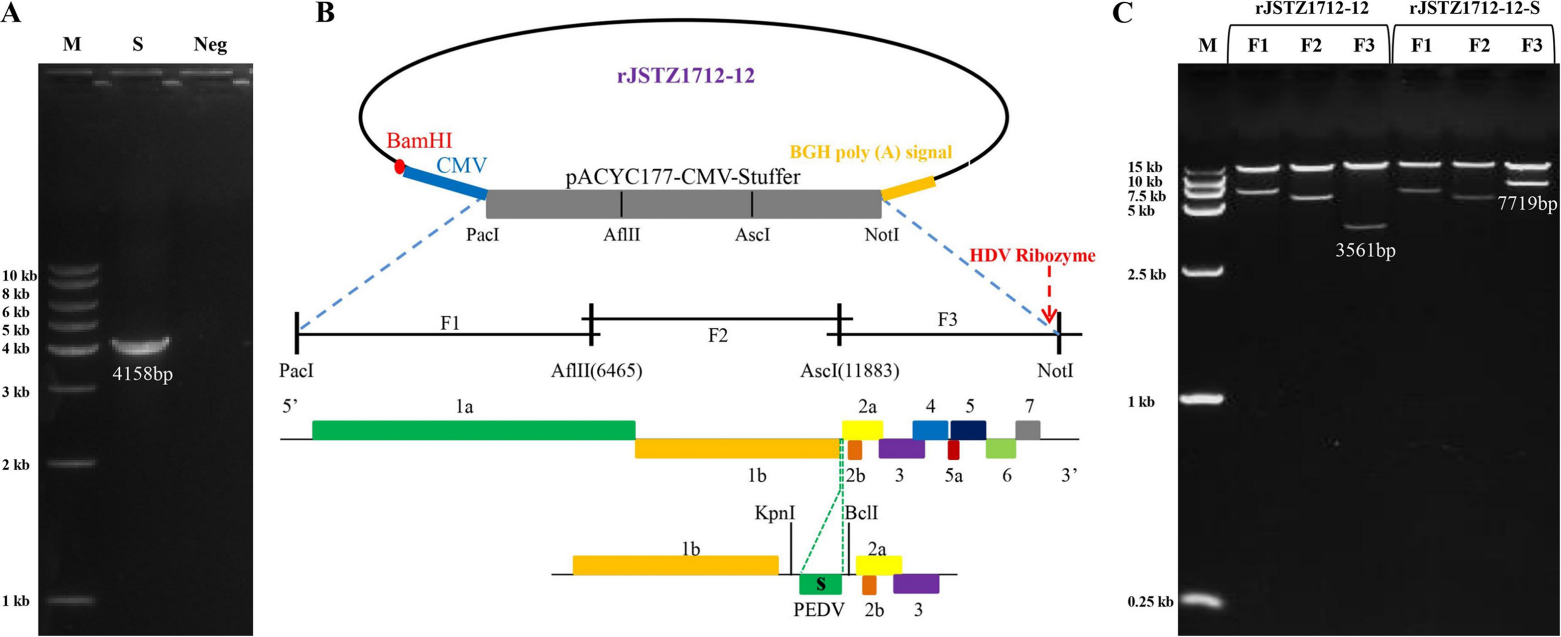
The chimeric rJSTZ1712-12-S virus was constructed using rJSTZ1712-12 infectious clone as the backbone. The amplicon of S gene of G2 PEDV XJ1904-34 strain is 4158 bp (A). The S gene of G2 PEDV XJ1904-34 strain was inserted between ORF1b and ORF2a genes of rJSTZ1712-12 (B). The rJSTZ1712-12 and rJSTZ1712-12-S recombinant plasmids were double digested with PacI*+AflII*, *AflII+AscI*, *AscI*+NotI to generate F1, F2 and F3 fragments, respectively. The size of rJSTZ1712-12-F3 (rJS-F3) is 3561 bp, while the size of rJSTZ1712-12-S-F3 (rJS-S-F3) with the inserted S gene is 7719 bp, indicating the successful construction of rJSTZ1712-12-S recombinant plasmid (C).

Firstly, S gene was amplified from G2 PEDV XJ1904-34 virus (Table 1), which is 4158 bp (GenBank accession number: OK642747) (Fig. 1A). Secondly, the S gene was inserted into the rJSTZ1712-12 clone as illustrated in Fig. 1B. Double restriction enzyme digestion showed that the F3-S fragment of rJSTZ1712-12-S is 7719 bp while the F3 fragment of rJSTZ1712-12 is 3561 bp, which supported the successful construction of rJSTZ1712-12-S (Fig. 1C). In addition, Sanger sequencing also confirmed the result (data not shown).

**TABLE 1:** PCR primers used for the construction of chimeric rJSTZ1712-12-S virus

No.	Primer	Sequence (5′–3′)^*a*^	Length (bp)
1	S-KpnI-fusion-F	***CTTTAGGCCTGAATTGAAGGTAC***CGCCACCATGAAGTCTTTAACCTACTTCTGGTTGT	58
2	S-BclI-fusion-R	***AGGGGTTGCCGCGGAATGATC***ATCACTGCACGTGGACCTTTTC	43

a The unique restriction enzyme sites are shown underlined. The homologous arms are highlighted in italic and bold.

**FIG 2:**
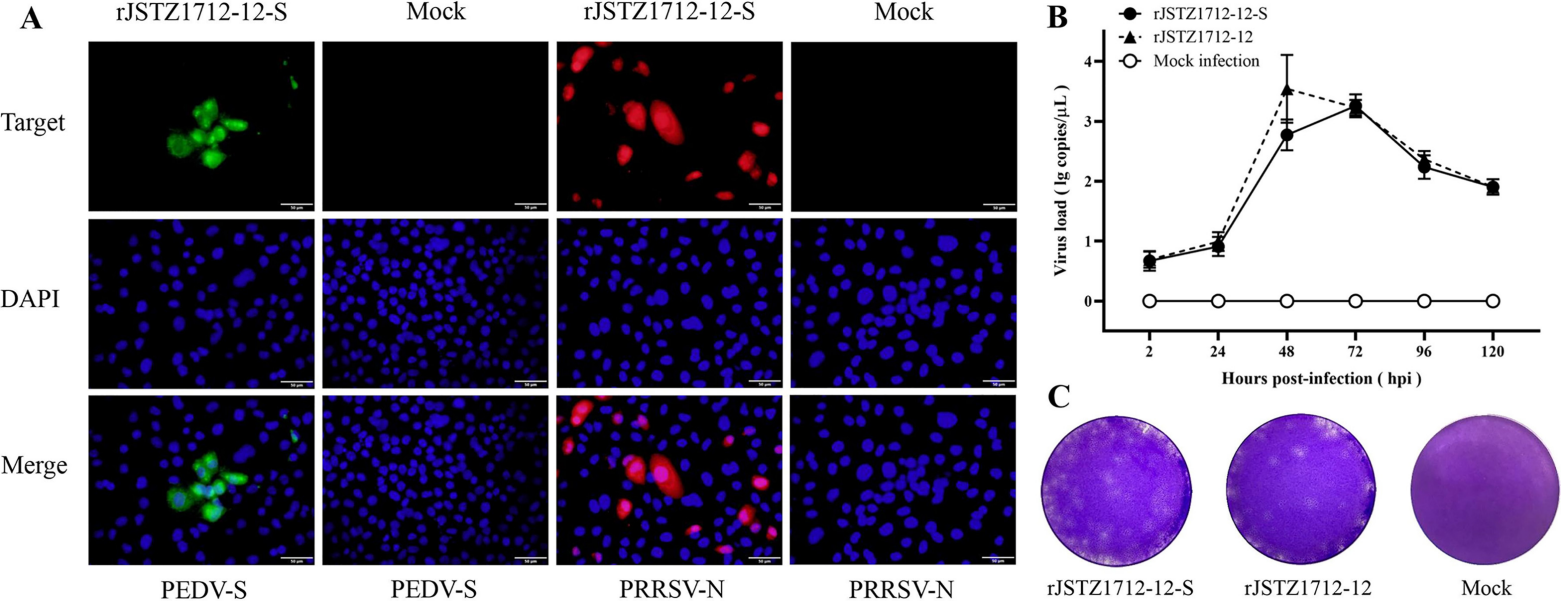
The chimeric rJSTZ1712-12-S virus was rescued in Marc-145 cells. IFA results showed that both PEDV S antigen and PRRSV N antigen could be detected in rJSTZ1712-12-S infected cells but not in mock infected cells (A). Multiple-step growth curves in Marc-145 cells within 120 hpi was determined by real-time RT-PCR, which showed that no significant difference was observed in the *in vitro* replication of parental rJSTZ1712-12 and chimeric rJSTZ1712-12-S viruses (B). The chimeric rJSTZ1712-12-S virus also has similar plaque morphology as the parental rJSTZ1712-12 virus (C).

During the infection of rJSTZ1712-12-S in Marc-145 cells, obvious cytopathic effects (CPE) could be observed at 72 hours postinfection (hpi). In addition, IFA showed that the expressions of PEDV S protein and PRRSV N protein can be detected in Marc-145 cells infected by rJSTZ1712-12-S but not in mock-infected cells (Fig. 2A). Multistep growth curves indicated that the growth kinetics of chimeric rJSTZ1712-12-S virus is similar (no significant difference, *P > *0.05) to the parental rJSTZ1712-12 virus (Fig. 2B). Moreover, plaque assay showed that rJSTZ1712-12-S and rJSTZ1712-12 viruses have similar plaque sizes (Fig. 2C). Therefore, these results supported the successful construction and rescue of chimeric rJSTZ1712-12-S virus, which has similar *in vitro* growth characteristics to the parental rJSTZ1712-12 virus.

### The chimeric rJSTZ1712-12-S virus is not pathogenic to piglets.

**FIG 3:**
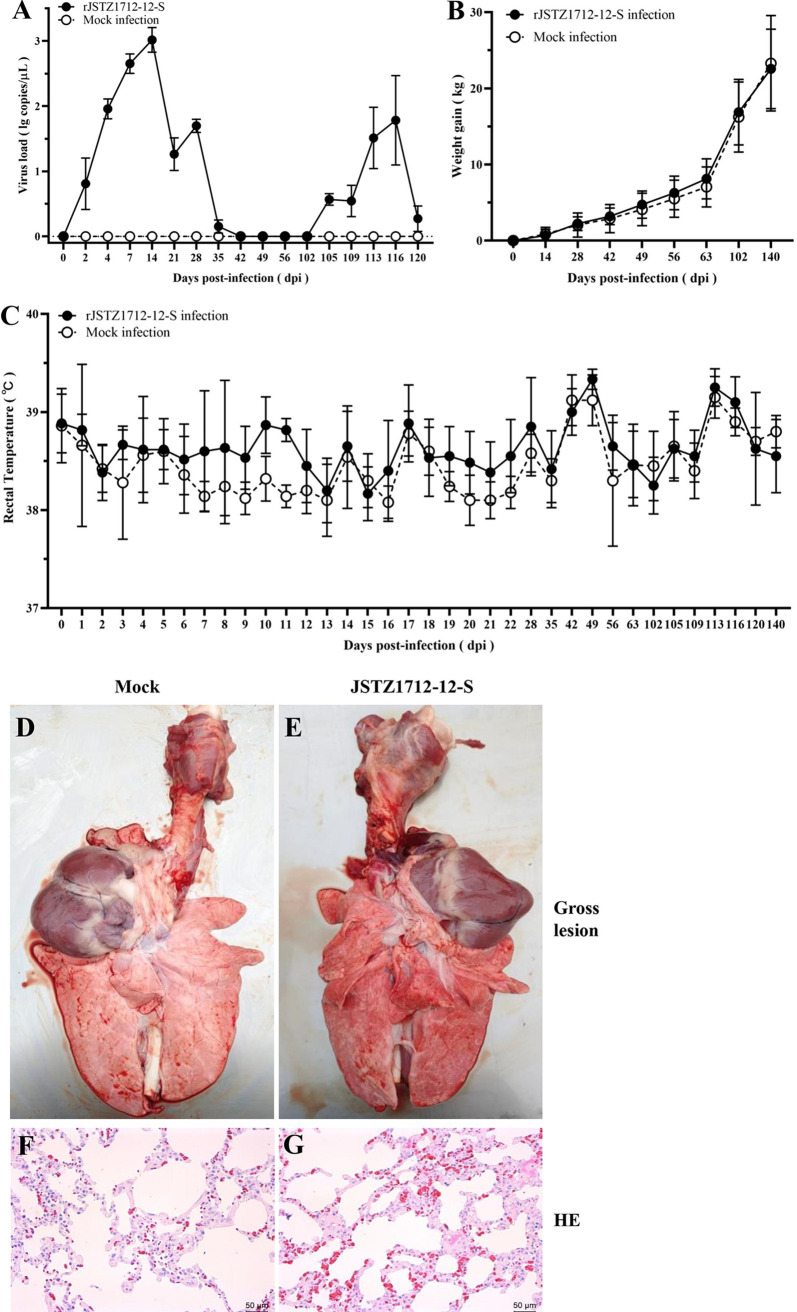
The chimeric rJSTZ1712-12-S virus is not pathogenic to piglets. Viremia could be generated with peaks at 14 dpi for both prime and boost inoculations in piglets (A). The inoculation of rJSTZ1712-12-S did not affect the weight gain of piglets (B). The inoculation of rJSTZ1712-12-S did not induce fever during the entire experiment (C). Necropsy examination did not detect obvious lung gross lesion in either rJSTZ1712-12-S infected or mock infected pigs (D and E). Only mild disseminated intravascular congestion could be observed in the lungs from rJSTZ1712-12-S infected pigs during the histopathological examination (F and G).

To evaluate the possibility of using rJSTZ1712-12-S as a vaccine candidate, pig inoculation was executed. Viremia could be detected from 2 days postinoculation (dpi) to 35 dpi since the prime immunization and from 3 dpi (105 dpi since the first infection) to 18 dpi (120 dpi since the first infection) after the boost inoculation (Fig. 3A). During the entire animal experiment, no clinical signs were observed in either rJSTZ1712-12-S-infected or mock-infected pigs. In addition, rJSTZ1712-12-S inoculation did not increase rectal temperature or affect weight gain (Fig. 3B and C). Necropsy observation showed that no obvious lung gross lesion was detected in either rJSTZ1712-12-S-infected or mock-infected pigs (Fig. 3D and E). Histopathological examination showed that only mild disseminated intravascular congestion was observed in the lungs from rJSTZ1712-12-S-infected pigs (Fig. 3F and G). These results suggested that rJSTZ1712-12-S has good *in vivo* replication efficacy, but it is not pathogenic to pigs.

### The chimeric virus induces shnAbs against both G2 PEDV and HP-PRRSV2.

**FIG 4:**
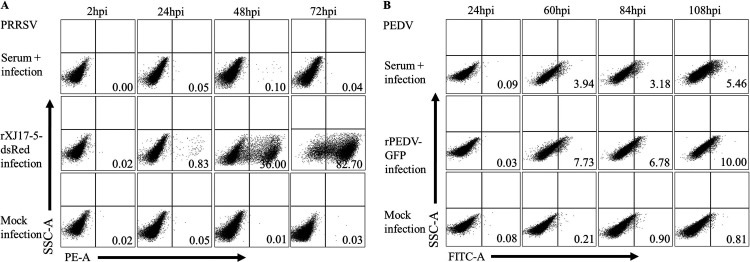
The chimeric rJSTZ1712-12-S virus induces shnAbs against both G2 PEDV and HP-PRRSV2. Flow cytometry results showed that no red fluorescent signals could be detected in serum (from rJSTZ1712-12-S immunized pigs) treated and infected samples, which indicated that rJSTZ1712-12-S-induced shnAbs could completely neutralize rXJ17-5-dsRed (A). Meanwhile, green fluorescent signals were significantly lower in serum treatment and infection cells than in rPEDV-EGFP infected cells (B). No specific fluorescent signals could be detected in mock infected cells.

To determine whether rJSTZ1712-12-S inoculation could induce protective immune responses against both PEDV and PRRSV, the generation of shnAbs against both viruses was determined by viral neutralization tests (Table 2). PEDV shnAbs could be detected as early as 22 dpi (one out of six pigs, 1/6) and reached a peak at 42 dpi (5/6). PRRSV shnAbs could be detected since 42 dpi (2/6), reached a peak at 63 dpi (4/6), and then decreased. Both PEDV and PRRSV shnAbs became undetectable in sera at 102 dpi. The highest shnAb titers could reach 1:32 for PEDV and 1:16 for PRRSV after prime inoculation. The neutralization activities of sera from rJSTZ1712-12-S immunized pigs against PEDV and PRRSV were further confirmed by flow cytometry. The results showed that red fluorescent signals could be detected in rXJ17-5-dsRed infected Marc-145 cells from 24 hpi (0.83%) to 72 hpi (82.70%) but not in the serum-treated infection cells or mock-infected cells (Fig. 4A; Fig. S1 in the supplemental material). From 60 hpi to 108 hpi, green fluorescent signals could be detected in rPEDV-EGFP infected Vero cells from 7.73% to 10.0% but obviously lower in the serum-added infection cells from 3.94% to 5.46% (Fig. 4B, Fig. S2). The incomplete neutralization of rJSTZ1712-12-S-induced shnAbs on rPEDV-EGFP is probably associated with relatively low S protein similarity (97.91%) between rPEDV-EGFP and XJ1904-34. The genome similarity between rJSTZ1712-12 and rXJ17-5 is 99.45%. Overall, all these results supported that rJSTZ1712-12-S immunization could induce shnAbs against both G2 PEDV and HP-PRRSV2 isolates.

**TABLE 2:** Systemic neutralizing antibodies induced by rJSTZ1712-12-S after prime-boost immunization

Days post infection (boost)	PRRSV neutralizing antibody responses (titer)^*a*^							PEDV neutralizing antibody responses (titer)^*b*^						
	Pig 6	Pig 7	Pig 8	Pig 9	Pig 10	Pig 11	Total	Pig 6	Pig 7	Pig 8	Pig 9	Pig 10	Pig 11	Total
Prime														
0	−^*c*^	−	−	−	−	−	0/6	−	−	−	−	−	−	0/6
22	−	−	−	−	−	−	0/6	−	−	−	−	−	+ (1:8)	1/6
42	−	−	−	+ (1:8)	+ (1:8)	−	2/6	+ (1:32)	+ (1:8)		+ (1:32)	+ (1:16)	+ (1:32)	5/6
63	+ (1:8)	−	+ (1:8)	+ (1:16)	+ (1:16)	−	4/6	−	−	−	+ (1:32)	+ (1:16)	+ (1:32)	3/6
86	+ (1:8)	−	−	+ (1:16)	+ (1:8)	−	3/6	−	+ (1:8)	−	−	−	+ (1:8)	2/6
Boost														
102 (0)	−	−	−	−	−	−	0/6	−	−	−	−	−	−	0/6
109 (7)	−	−	−	−	+ (1:8)	−	1/6	−	−	−	−	−	+ (1:8)	1/6
116 (14)	+ (1:8)	−	−	−	+ (1:16)	+ (1:8)	3/6	+ (1:8)	−	+ (1:8)	−	+ (1:8)	+ (1:8)	4/6
126 (24)	+ (1:8)	+ (1:8)	+ (1:8)	+ (1:8)	+ (1:16)	+ (1:8)	6/6	+ (1:8)	+ (1:16)	+ (1:8)	+ (1:16)	+ (1:16)	+ (1:32)	6/6
140 (38)	+ (1:16)	+ (1:8)	+ (1:16)	+ (1:16)	+ (1:32)	+ (1:16)	6/6	+ (1:16)	+ (1:32)	+ (1:32)	+ (1:16)	+ (1:16)	+ (1:64)	6/6

a The PRRSV neutralizing activities were evaluated in Marc-145 cells using parental HP-PRRSV2 JSTZ1712-12 isolate.

b The PEDV neutralizing activities were evaluated in Vero cells using homologous G2 PEDV YC2014 isolate.

c −, indicates no neutralizing antibody production; +, indicates the production of neutralizing antibody and the titer is shown within the bracket.

### A secondary immunization rapidly boosts shnAb responses.

To estimate the protective memory humoral immune responses, a boost immunization was performed at 102 dpi when rJSTZ1712-12-S-induced shnAbs were completely degraded. After boost immunization, shnAbs could be induced as early as 7 days post boost (dpb) for both PEDV (1/6) and PRRSV (1/6). Furthermore, PEDV and PRRSV shnAbs could be detected in all immunized pigs since 24 dpb (126 dpi). The highest shnAb titers could reach 1:64 for PEDV and 1:32 for PRRSV after boost immunization (Table 2). However, the neutralization activities against other heterologous PEDV or PRRSV strains were significantly decreased or undetectable (data not shown). In addition, the IFN-γ levels were nearly undetectable in the sera during the entire experiment (Fig. S3). These results indicated that PEDV and PRRSV shnAbs can be rapidly induced after boost immunization of the chimeric rJSTZ1712-12-S virus.

### Passive transfer of shnAbs confers insufficient protection against dual challenge of high virulent G2 PEDV and HP-PRRSV2 strains.

**FIG 5:**
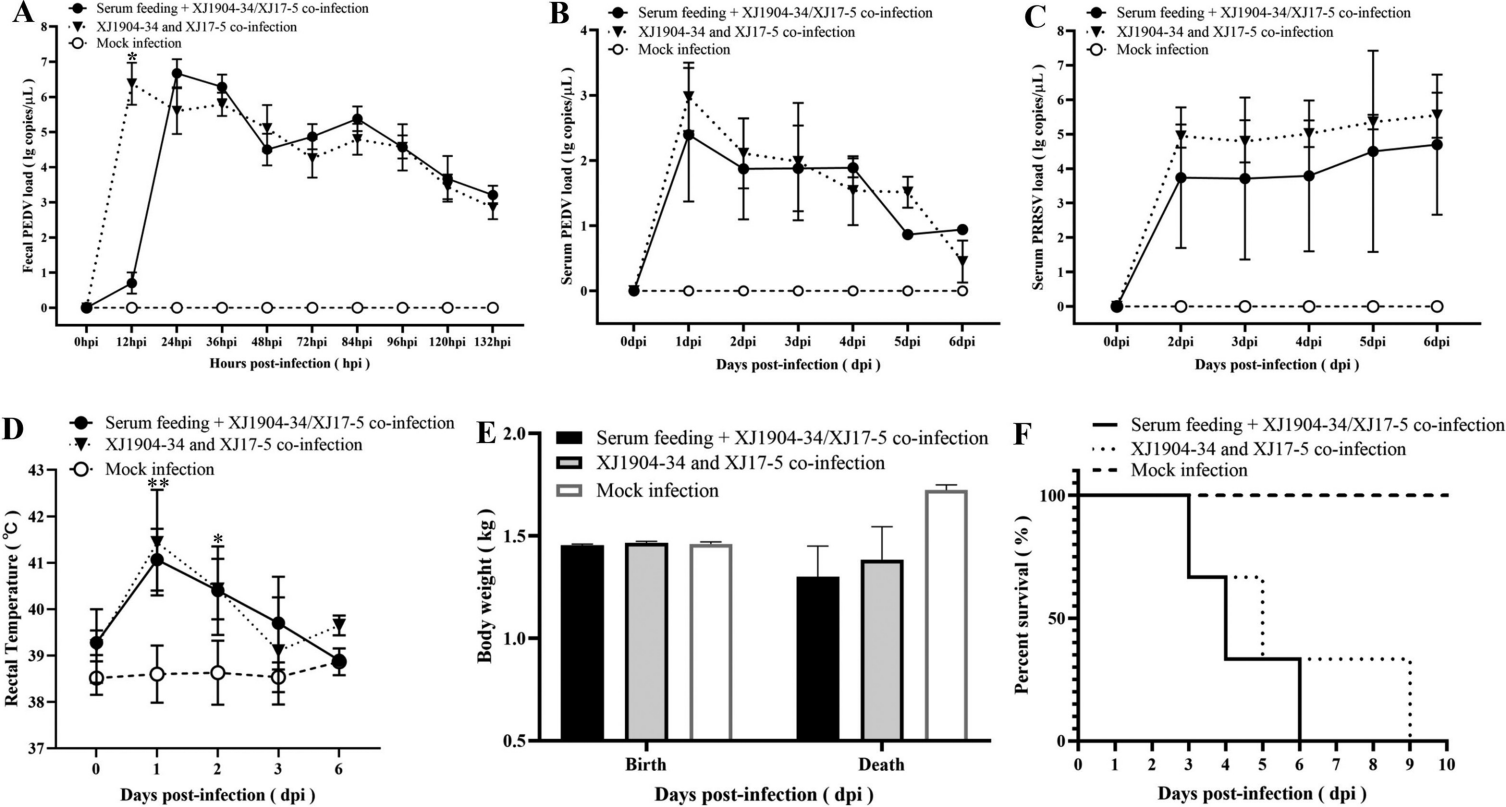
The chimeric rJSTZ1712-12-S virus induced shnAbs provide insufficient protection against dual challenge of high pathogenic G2 PEDV XJ1904-34 strain and HP-PRRSV2 XJ17-5 isolate in neonatal piglets. PEDV virus load in feces was significantly lower in the serum feeding/challenge group than in the challenge group at 12 hpi (A). PEDV viremia was relatively lower in the serum feeding/challenge group than in the challenge group at 1 dpi but no difference in the following days (B). PRRSV viremia was always relatively lower in the serum feeding/challenge group than in the challenge group from 2 dpi to 6 dpi but with no statistically significant difference (*P > *0.05) (C). Dual challenge induced high fever in neonatal piglets of both the serum feeding/challenge group and the challenge group at 1 and 2 dpi but not in the mock infected group (D). Neonatal piglets in both the serum feeding/challenge group and the challenge group had a certain degree of weight loss at the end of this study (*P > *0.05) (E). All neonatal piglets in the serum feeding/challenge group and the challenge group died from 3 dpi to 9 dpi (F).

To evaluate whether the rJSTZ1712-12-S-induced shnAbs could provide protection against dual challenge of high virulent PEDV and PRRSV isolates, neonatal piglets deprived of sow colostrum were fed with mixed sera containing shnAbs (1:32 for PEDV and 1:16 for PRRSV) and challenged with highly pathogenic G2 PEDV XJ1904-34 virus and HP-PRRSV2 XJ17-5 isolate. PEDV virus loads in piglets of sera-feeding/challenge group were significantly lower than the challenge group in the feces at 12 hpi (*P < *0.05) (Fig. 5A) and relatively lower in the sera at 1 dpi (*P > *0.05) (Fig. 5B), indicating that rJSTZ1712-12-S-induced shnAbs did reduce the replication of G2 PEDV at a very early stage of infection, but this did not last in the following days. Even though no significant difference (*P > *0.05) was detected due to high discrepancy in different piglets, PRRSV virus loads in piglets of the sera-feeding/challenge group were lower than the those of the challenge group from 2 dpi to 6 dpi (Fig. 5C), suggesting that rJSTZ1712-12-S-induced shnAbs also likely decrease the proliferation of HP-PRRSV2 in neonatal piglets.

**FIG 6:**
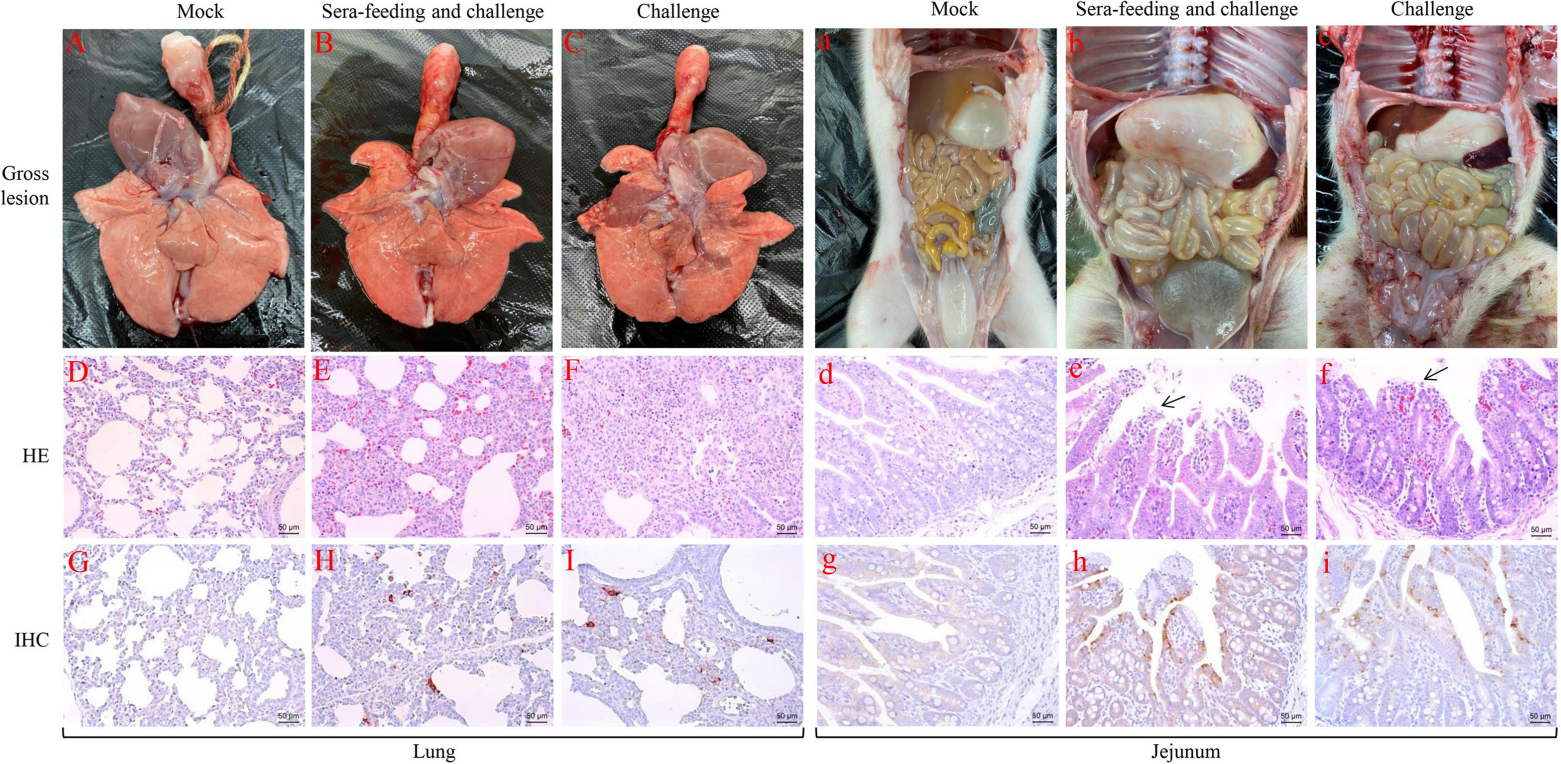
Neonatal piglets in the serum feeding/challenge group seemed to die of PEDV infection rather than PRRSV infection. Obvious lung consolidation could be observed in the challenge group but not in the serum feeding/challenge group or the mock infected group (A–C). Mild to severe degrees of widened alveolar septum were observed in the sera-feeding/challenge group and the challenge group, respectively (D–F). PRRSV specific antigen could be detected in both the sera-feeding/challenge group and the challenge group (G–I). Obvious flatulence and thinning of intestinal walls could be observed from both the sera-feeding/challenge group and the challenge group (a–c). Exfoliation of mucosal epithelial cells and exposed lamina propria of jejunum could be detected in both the sera-feeding/challenge group and the challenge group (d–f). PEDV specific antigen could also be detected in jejunum of both the sera-feeding/challenge group and the challenge group (g-i).

However, compared with the negative control group, neonatal piglets in both the sera-feeding/challenge group and challenge group showed high fever at 1–2 dpi (Fig. 5D) and weight loss at the end of this study (Fig. 5E). In addition, neonatal piglets in the challenge group suffered diarrhea and vomiting after 12 hpi, the same as the sera-feeding/challenge group after 18 hpi, and all piglets in these two groups died between 3 dpi and 9 dpi (Fig. 5F). Necropsy examination observed severe lung consolidations in the challenge group but not in the sera-feeding/challenge or mock infected groups (Fig. 6A to C). However, obvious flatulence and thinning of intestinal walls could be observed from both the sera-feeding/challenge group and the challenge group but not from the negative control group (Fig. 6a to c). Histopathological examination identified mild to severe degrees of widened alveolar septum in the sera-feeding/challenge group and the challenge group, respectively (Fig. 6D to F). However, exfoliation of mucosal epithelial cells and exposed lamina propria of jejunum could be detected in both the sera-feeding/challenge group and the challenge group (Fig. 6d to f). During the immunohistochemical examination, PRRSV antigens could be detected in the lung, and PEDV antigens could be found in the jejunum of both sera-feeding/challenge piglets and challenge piglets but not in mock-infected piglets (Fig. 6G, I, g to i). These results indicated that rJSTZ1712-12-S-induced shnAbs can partly inhibit the replication of G2 PEDV and HP-PRRSV2, especially at the very early stage, but cannot confer enough protection for neonatal piglets against the dual challenge of highly pathogenic G2 PEDV and HP-PRRSV2 isolates. In addition, gross and histopathological examinations suggested that the death of neonatal piglets in the sera-feeding/challenge group seems to be more associated with PEDV infection rather than PRRSV infection. Overall, these results supported that shnAbs confer insufficient protection against PEDV and PRRSV coinfection and are not suitable for the protective efficacy evaluation of PED and PRRS bivalent vaccine, especially for the PED vaccine protective efficacy.

## DISCUSSION

MLV immunization is a commonly used strategy for combatting PED and PRRS in China. However, both PED and PRRS commercial vaccines have some limitations. For example, current PED vaccines are insufficiently protective against the prevalent G2 PEDV isolates. Commercial PRRS vaccines provide satisfied protection against homologous strains but not for heterologous isolates. In addition, current MLVs also have safety issues. Moreover, different types of PED and PRRS MLVs are widely used in China’s swine herds, resulting in huge immune pressures. The coinfection of PEDV and PRRSV occurs commonly in China, but no commercial vaccine can provide dual protection against the prevalent PEDV and PRRSV isolates. Therefore, we developed a chimeric HP-PRRSV2 strain expressing S protein of G2 PEDV and evaluated the protective efficacy of rJSTZ1712-12-S-induced shnAbs against the dual challenge of high virulent G2 PEDV and HP-PRRSV2 isolates in this study.

Reverse genetics have been utilized to improve the efficacy and safety of PRRSV vaccines (34). For example, they have been used to improve heterologous protection by DNA shuffling or carrying a consensus sequence (29, 35), to attenuate PRRSV by codon pair de-optimization (36) and to develop DIVA vaccines by removing B cell epitopes in nsp2 and M proteins (37). In addition, a PRRSV infectious clone has been used as a viral vector for the expression of PCV2 capsid and swine influenza HA protein (31, 32). PEDV S protein has been expressed in VSV, TGEV, baculovirus, swinepox, and parapoxvirus, which could induce PEDV shnAbs and was reported to provide satisfied homologous protection (8, 27, 38–40). In this study, a chimeric HP-PRRSV2 strain expressing S protein of G2 PEDV was constructed, which could induce shnAbs against both G2 PEDV and HP-PRRSV2. However, the shnAbs provided insufficient protection against the dual challenge of highly pathogenic homologous G2 PEDV strain and HP-PRRSV2 isolate in neonatal piglets. Noticeably, a recent study showed that piglets immunized with a spike subunit vaccine could also induce high levels of PEDV shnAbs, but it resulted in more severe disease symptoms and enhanced virus replication when exposed to PEDV challenge (41). These observations indicated that shnAbs are inadequate for the evaluation of PEDV vaccine efficacy.

In this study, we evaluated the dual protection against both high virulent G2 PEDV and HP-PRRSV2 conferred by rJSTZ1712-12-S, which has not been explored in previous studies. Both PEDV and PRRSV are prevalent, and the coinfection has commonly occurred in China. In addition, the coinfection of PEDV and PRRSV has a greater effect on the reduction of feed intake and pig growth rates than each single infection (42, 43). According to previous studies, shnAbs induced by chimeric viruses expressing S protein of PEDV could provide satisfactory protection against the single challenge of homologous PEDV strains (8, 38, 39). However, this study showed that even though rJSTZ1712-12-S could also induce PEDV shnAbs, it could not protect neonatal piglets against the challenge of parental high virulent XJ1904-34, which suggested that coinfection with PRRSV might influence the neutralization activity of shnAbs on the parental G2 PEDV strain. Vice versa, the coinfection with PEDV might also influence the protective efficacy of shnAbs against the homologous high virulent HP-PRRSV2 XJ17-5 isolate. Considering the oral feeding of shnAbs and the oral/intranasal challenge of XJ17-5, another potential reason for insufficient protection against XJ17-5 by shnAbs is that passively-fed shnAbs would unlikely migrate through the common mucosal system and reach the respiratory surface to confer protection against homologous HP-PRRSV2.

In addition, to evaluate the correlation between PEDV and PRRSV shnAbs and vaccine protective efficacy, we challenged neonatal piglets deprived of sow colostrum to evaluate the protective efficacy in this study. Several previous studies showed complete/satisfied protection conferred by PEDV-S-induced shnAbs when the piglets (3–5 weeks old) were immunized by the corresponding recombinant viruses and challenged at 21–60 dpi (8, 38, 39). G2 PEDV mainly causes severe clinical symptoms and high mortality in suckling piglets but not in growth-fattening pigs. Therefore, our results would indicate that whether the shnAbs induced by previous reported chimeric viruses could still confer satisfied protection in newborn piglets needs further evaluation.

Moreover, we directly evaluated the protective efficacy of shnAbs via serum passive transfer rather than immunization of sows in this study. A previous study showed that immunization of recombinant VSV expressing S protein of G2b PEDV in pregnant sows could provide robust protective lactogenic immunity to neonatal piglets against homologous virulent PEDV challenge (27). Passive lactogenic immunity is the most promising and effective way to protect neonatal piglets against PEDV infection (44). Protection of neonatal piglets via lactogenic immunity depends on the trafficking of PEDV-specific IgA plasmablasts to the mammary gland and accumulation of secretory IgA (sIgA) in colostrum. Even though shnAbs are commonly used to evaluate the vaccine protective efficacy, no direct evidence supports the correlation between serum shnAbs and protective efficacy against the onsets of swine enteric virus infections (45, 46). PEDV shnAbs might even be related to the enhancement of PEDV replication and disease symptoms in piglets (41). Our results showed that rJSTZ1712-12-S-induced shnAbs could reduce PRRSV-associated lung lesions in neonatal piglets but did not alleviate PEDV-associated intestinal lesions, which suggested that the death of neonatal piglets was more likely associated with PEDV challenge in this study. Therefore, whether immunization of pregnant sows with rJSTZ1712-12-S could induce intestinal nAbs (sIgA) in milk and confer higher protective efficacy in neonatal piglets needs further investigation.

Additionally, the insufficient protection might be also associated with the high virulence of both G2 PEDV and HP-PRRSV2 isolates, both of which could cause 100% mortality in suckling piglets, Or it might be related to the high number of viruses used in our challenge study. Similar amounts of both viruses were used for neonatal piglets in this study as previous described for 6–12-week-old pigs (8, 38, 39). Or it might be correlated with the lack of IFN-γ secretion induced by rJSTZ1712-12-S, because protective cellular immunity generally also plays an important antiviral role. Or it might be associated with relatively low titer of shnAbs (1:32 for PEDV and 1:16 for PRRSV) used in the passive transfer study. The used shnAbs might be significantly degraded across the gastric and intestinal digestion tracts (47), which could result in even lower titers of shnAbs *in vivo*. In addition, the low titer of shnAbs after prime-boost inoculation might be also associated with the use of 3-week-old piglets. The existence of maternal immunity and the pre-mature developing immune system in 3-week-old piglets might interfere with vaccination effects. We used the available 3-week-old piglets in the vaccination study because it has been extremely difficult to obtain negative pigs for PRRSV, PEDV, and other major pathogens such as ASFV in recent years due to high prevalence of PRRS and the outbreaks of ASF in China in 2018. For similar reasons, it is not only more difficult to obtain PRRSV negative sows but also more expensive to use pregnant sows in the immunization study.

In conclusion, this study provides the first evidence that shnAbs confer insufficient protection against PEDV and PRRSV coinfection and are inadequate for the protective efficacy evaluation of PED and PRRS bivalent vaccine candidates.

## MATERIALS AND METHODS

### Viruses and cells.

Viruses used in this study were all stored in our laboratories, including G2 PEDV XM1-2 strain (KX812523) (2), YC2014 isolate (KU252649) (48), XJ1904-34 virus (OK642747), rPEDV-EGFP infectious clone and G1 CV777 strain, PRRSV2 CH-1a-like SD1612-1 isolate, VR2332-like JSYC20-05-1 isolate, NADC30-like SD17-36 isolate, HP-PRRSV2 XJ17-5 and JSTZ1712-12 strains, rJSTZ1712-12 and rXJ17-5-dsRed infectious clones (3, 49–51), and PRRSV1 HLJB1 isolate (52). Vero cell, Marc-145 cells, and BHK-21 cells were cultured in Dulbecco minimum essential median (DMEM) supplemented with 10% fetal bovine serum (FBS) and antibiotics.

### Construction of a chimeric HP-PRRSV2 expressing S protein of G2 PEDV.

To construct a chimeric virus expressing S protein of G2 PEDV, the previously developed HP-PRRSV2 infectious clone rJSTZ1712-12 was used as the backbone (3). At first, the S gene of G2 PEDV was amplified from the currently prevalent XJ1904-34 strain using high fidelity 2 × Phanta Flash Master Mix (Vazyme Biotech Co., Ltd., China) with primers shown in Table 1. Secondly, the S gene was inserted between ORF1b and ORF2a genes of the rJSTZ1712-12 infectious clone as we previously described (33, 51). In addition, the recombined plasmid rJSTZ1712-12-S was confirmed by double restriction enzyme digestion (PacI*+AflII*, *AflII+AscI*, *AscI*+NotI) and Sanger sequencing.

### Rescue of the chimeric virus.

The obtained rJSTZ1712-12-S full-length clone was transfected into BHK-21 cells using Lipofectamine 3000 Reagent (Invitrogen, USA) and cell culture supernatant obtained at 48 h posttransfection (hpt) was serially passaged on Marc-145 cells. The successful rescue of rJSTZ1712-12-S infectious virus was confirmed by indirect immunofluorescence assay (IFA). PEDV S expression was detected using PEDV S2-specific murine MAb Anti-S2 as the primary antibody and DyLight 488 (Thermo Fisher, USA) as the secondary antibody, while PRRSV N expression was also detected using PRRSV anti-N murine MAb IC9C8 as the primary antibody and DyLight 594 (Invitrogen, USA) as the secondary antibody (50, 53). In addition, to determine the growth kinetics of rJSTZ1712-12-S *in vitro*, Marc-145 cells were infected with 100 median tissue culture infectious doses (TCID_50_) of rJSTZ1712-12-S and rJSTZ1712-12 viruses, respectively. The multiple-step growth curves within 120 h postinfection (hpi) were determined by PRRSV universal real-time RT-PCR assay (15). The plaque morphology was also determined in Marc-145 cells as previously described (26).

### Piglet inoculations.

To evaluate the safety and immunogenicity of rJSTZ1712-12-S, piglet inoculation was performed. All animal experiments in this study were approved by the Animal Welfare and Ethic Committee of Yangzhou University with reference number 202104002. Eleven 3-week-old PEDV and PRRSV free piglets (a litter) were randomly divided into two groups. Six piglets in the first group were intramuscularly and intranasally inoculated with 2 mL 10^5.0^ TCID_50_/mL rJSTZ1712-12-S (5th passage), while the five piglets in the second group were inoculated with 2 mL DMEM to serve as the negative control. At 102 days postinoculation (dpi) (shnAbs were undetectable in sera of all pigs), the first group pigs were injected with rJSTZ1712-12-S again to boost the protective immunity.

Clinical signs were monitored daily and the body weight was measured biweekly. Rectal temperature was regularly recorded especially within 3 weeks after the prime and boost inoculations. Sera were collected weekly for the analyses of virus load and shnAb level. The dynamics of viremia were detected by PRRSV real-time RT-PCR assay (15). The sera were submitted to virus neutralization test against homologous G2 PEDV YC2014 strain in Vero cells and against parental HP-PRRSV2 JSTZ1712-12 in Marc-145 cells that both induce obvious cytopathic effects (CPE) (54). The absence of CPE at a 1:8 dilution of serum was considered as positive for the presence of shnAbs. In addition, the neutralization activities of sera on G2 PEDV (rPEDV-EGFP) and HP-PRRSV2 (rXJ17-5-dsRed) were further evaluated by a FACSVerse flow cytometer (BD Biosciences, USA) as previously described (51). Moreover, IFN-γ in the sera was detected using the commercial Porcine IFN-gamma ELISA Kit (ABSIN, Beijing, China). The pigs were euthanized at 140 dpi and sera containing shnAbs were collected for passive transfer study. Tissue samples including lungs and lymph nodes were submitted to histopathological examination for safety evaluation (12).

### Passive transfer study.

Generally, PEDV causes high mortality and severe diseases in neonatal piglets but not in adult pigs. Immunization of sows to provide protective lactogenic immunity is the most common practice for preventing PEDV infection in suckling piglets. Therefore, to evaluate the realistic protection efficacy of shnAbs induced by rJSTZ1712-12-S, we performed passive transfer and challenge study in neonatal piglets rather than directly challenge the immunized growth-fattening pigs. Sera collected from rJSTZ1712-12-S inoculated pigs at 140 dpi were pooled and used to feed newborn piglets deprived of the colostrum. Nine newborn piglets (a litter) were randomly divided into three groups (three per group). The sera were added to milk at a ratio of 50 mL of serum per L of milk. Each piglet was fed with ∼25 mL milk or serum-milk solution eight times a day (40). First group piglets were fed with the serum-milk solution and then orally and intranasally dual challenged with 1 mL 10^5.5^ TCID_50_ virulent G2 PEDV XJ1904-34 strain and 1 mL 10^5.0^ TCID_50_ virulent HP-PRRSV2 XJ17-5 isolate at the second day (48, 50). Second group piglets were fed with pure milk and challenged with XJ1904-34 and XJ17-5 the same as the first group. Third group piglets were fed with the serum-milk solution without challenge. Piglets were monitored daily for 10 days. Anal and oral swabs were collected from each pig daily and submitted to PEDV and PRRSV detection by real-time RT-PCR assays (2, 15). Jejunums and lungs were collected from dead or euthanized piglets for histopathological and immunohistochemical examinations.

### Statistical analysis.

The data of virus load, rectal temperature, and weight gain in this study are shown as means ± standard deviations (SD). The differences between groups were determined by Mann-Whitney U test using GraphPad Prism 8 XML project as we previously described (55). A *P* value < 0.05 was determined as statistically significant.

### Ethics statement.

All animal experiments in this study were approved by the Animal Welfare and Ethic Committee of Yangzhou University with the reference number of 202104002.
