# Supplementary material for: Systemic Homologous Neutralizing Antibodies Are Inadequate for the Evaluation of Vaccine Protective Efficacy against Coinfection by High Virulent PEDV and PRRSV
Source: Microbiol Spectr. 2022 Mar 22;10(2):e02574-21. doi: 10.1128/spectrum.02574-21 (PMC9045284; doi:10.1128/spectrum.02574-21)
Supplement: SUPPLEMENTAL FILE 1 — Supplemental material. Download SPECTRUM02574-21_Supp_1_seq9.pdf, PDF file, 0.6 MB [file spectrum02574-21_supp_1_seq9.pdf]

Figure S1

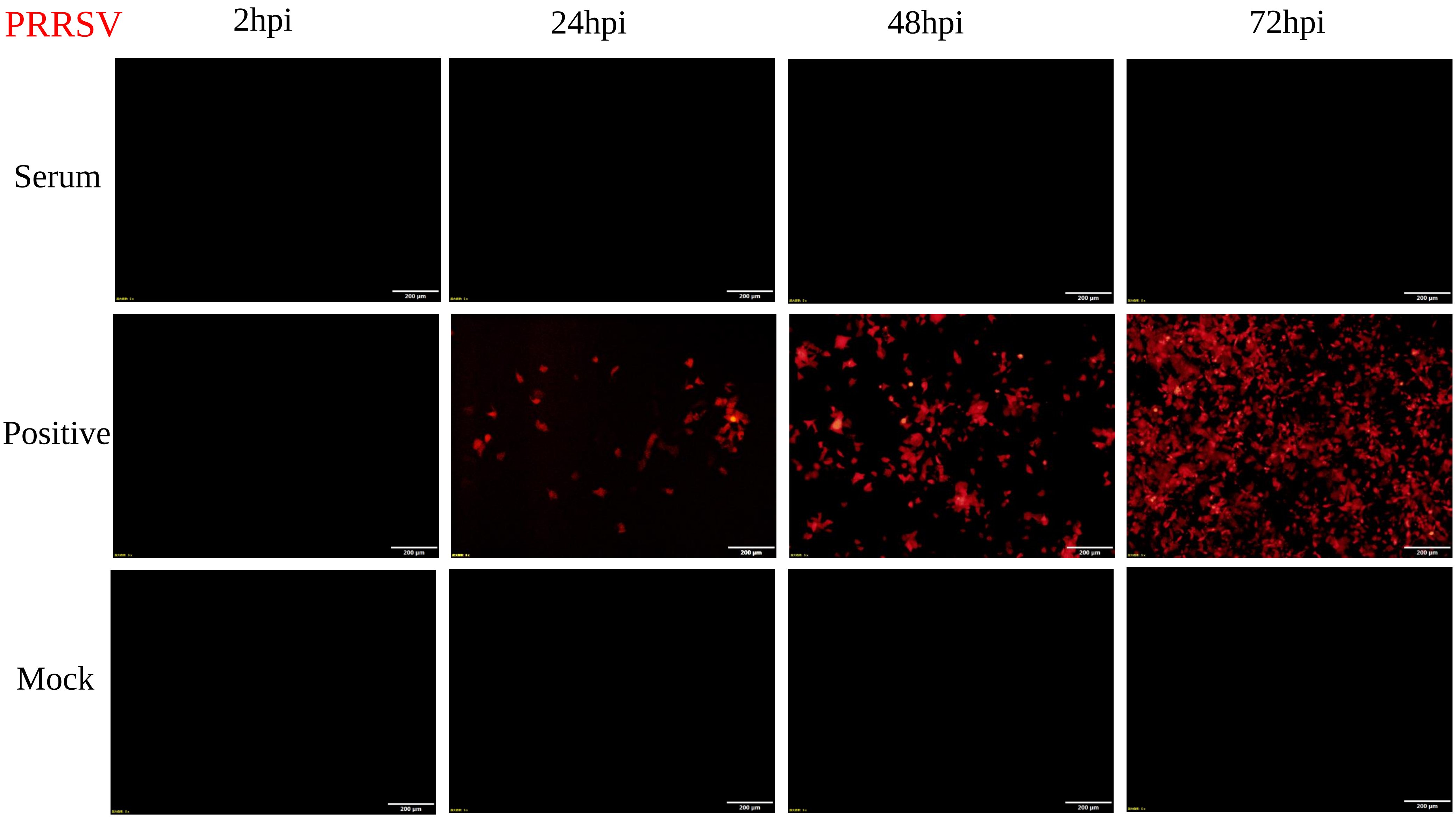

Figure S2

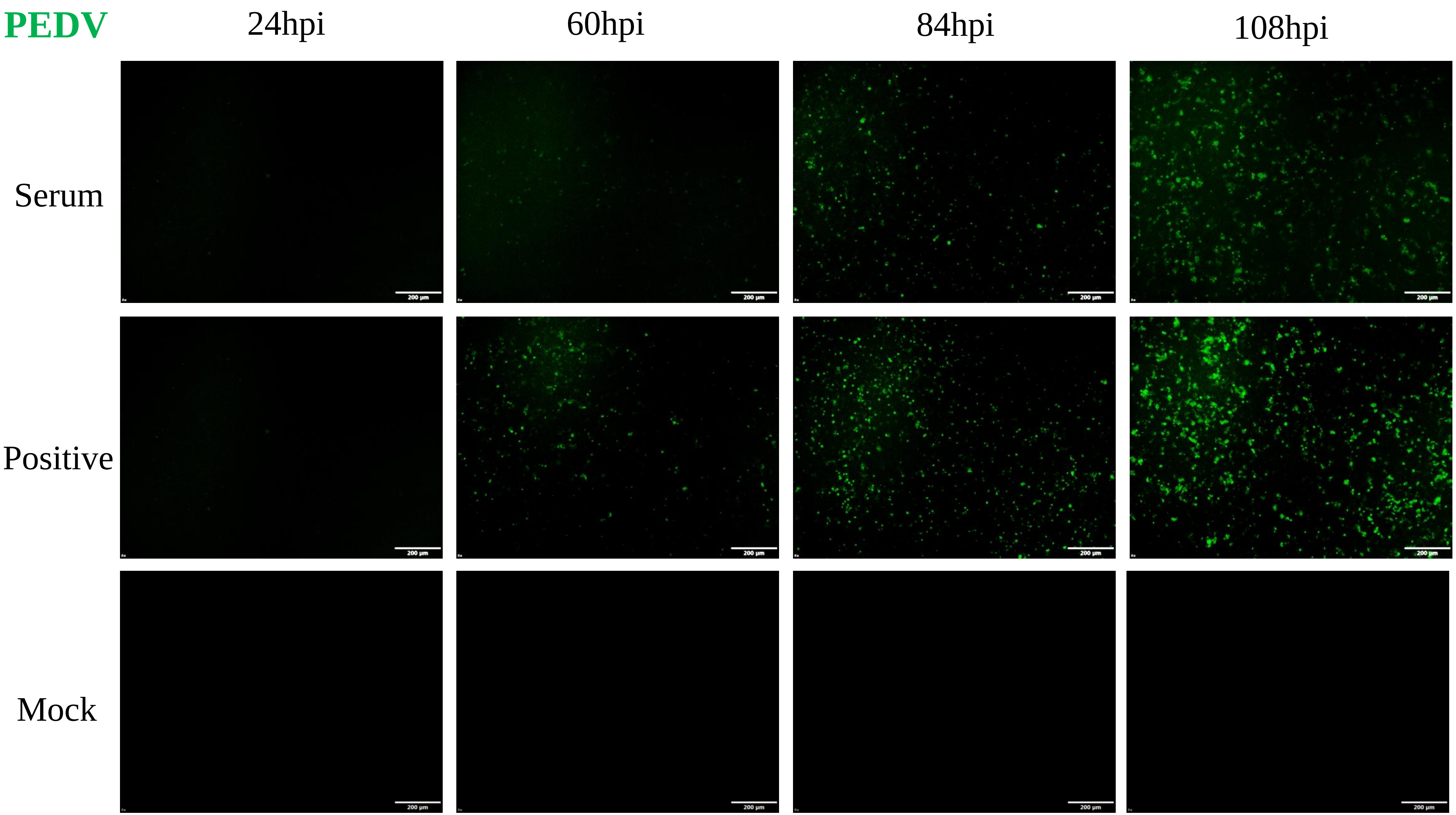

Figure S3

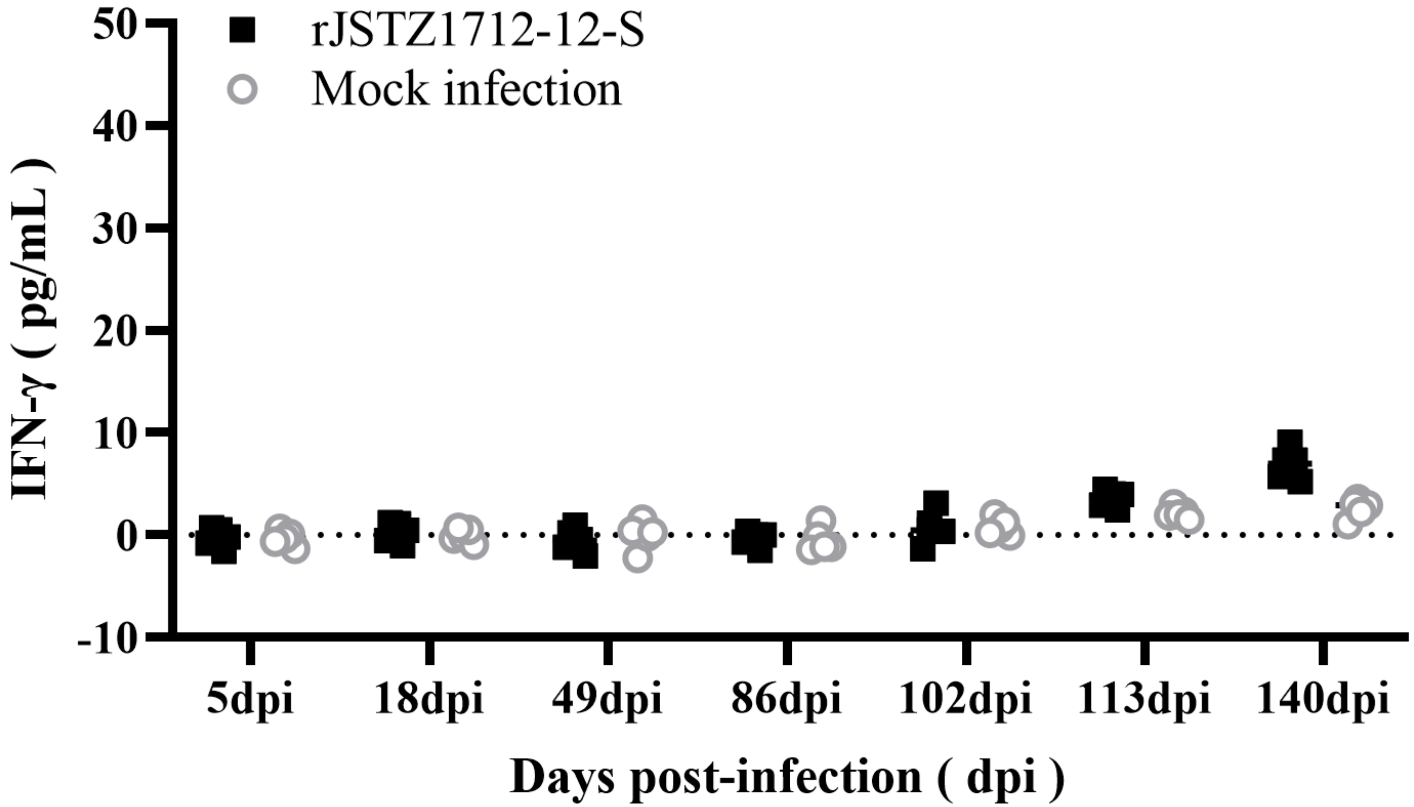
rJSTZ1712-12-S
Mock infection
IFN-γ (pg/mL)
50
40
30
20
10
0
-10
5dpi
18dpi
49dpi
86dpi
102dpi
113dpi
140dpi
Days post-infection (dpi)

FIGURE LEGENDS FOR SUPPLEMENTARY MATERIALS

Figure S1. The chimeric rJSTZ1712-12-S virus induced shnAbs can completely neutralize rXJ17-5-dsRed in Marc-145 cells. During the rXJ17-5-dsRed infection (100 $TCID_{50}$) in Marc-145 cells, red fluorescent signals were gradually increased from 0.83% to 82.70% from 24 hpi to 72 hpi. However, in serum (with 1:16 PRRSV shnAbs) treated and infected cells or mock infected cells, no red fluorescent signals could be detected from 0 hpi to 72 hpi.

Figure S2. The chimeric rJSTZ1712-12-S virus induced shnAbs can significantly decrease the replication of rPEDV-GFP in Vero cells. During the rPEDV-GFP infection (100 $TCID_{50}$) in Vero cells, obviously lower green fluorescent signals were detected in serum (with 1:16 PEDV shnAbs) treated and infected cells than in rPEDV-GFP infected cells from 60 hpi (3.94% vs 7.73%) to 108 hpi (5.46% vs 10.00%).

Figure S3. The chimeric rJSTZ1712-12-S virus could not induce the secretion of IFN-γ during the prime-boost inoculation. The IFN-γ levels were nearly undetectable in the sera from both rJSTZ1712-12-S infected and mock infected piglets from 0 dpi to 140 dpi.
